# Lipid Nanoparticle-Encapsulated PolyI:C as an Adjuvant Enhances Both Humoral and Cellular Immune Responses to the Hepatitis B Vaccine

**DOI:** 10.3390/vaccines14050397

**Published:** 2026-04-29

**Authors:** Zhixian Zhao, Bin Wang, Hao Wang, Qiang Zhang, Yunfei Liang, Yuan Liu

**Affiliations:** 1 LakeShore Biopharma (Beijing) Co., Ltd., Beijing 100176, Chinahao.wang@lakeshorebio.com (H.W.); 2 Liaoning Yisheng Biopharmaceutical Co., Ltd., Shenyang 110136, Chinaqiang.zhang@lakeshorebio.com (Q.Z.); yunfei.liang@lakeshorebio.com (Y.L.)

**Keywords:** lipid nanoparticle, polyinosinic-polycytidylic acid, HBsAg, adjuvant, immunogenicity

## Abstract

**Background**: Currently marketed hepatitis B vaccines are primarily recombinant protein vaccines. However, their antigen immunogenicity is relatively weak, requiring combination with effective adjuvants to enhance the immune response. The development of novel, highly effective adjuvants is a key strategy for optimizing vaccine performance. Polyinosinic-polycytidylic acid (PolyI:C), a synthetic double-stranded RNA analog, activates TLR3/RLR pathways to enhance T-cell priming and cellular immunity. However, its utility as a sole adjuvant is limited by rapid nuclease degradation and poor cytosolic delivery. Lipid nanoparticles (LNPs), a mature delivery platform, enable high encapsulation efficiency, efficient cellular uptake, and endosomal escape. **Objectives**: This study aimed to evaluate the adjuvant effect of LNP-encapsulated PolyI:C (LNP-PolyI:C) on the immunogenicity of hepatitis B surface antigen (HBsAg) in vivo. **Methods**: The colloidal stability of LNP-PolyI:C stored at 2–8 °C for 9 months was monitored using dynamic light scattering (DLS) on a Zetasizer Lab instrument. Serum levels of HBsAg-specific IgG, IgG1, and IgG2a antibodies in immunized Kunming mice were measured by enzyme-linked immunosorbent assay (ELISA). The secretion of HBsAg-specific cytokines by splenocytes was analyzed using flow cytometry and enzyme-linked immunospot (ELISpot) assay. **Results**: The results demonstrated that the LNP-encapsulated PolyI:C adjuvant significantly increased the secretion of HBsAg-specific IFN-γ, IL-2, and TNF-α by splenocytes, indicating a Th1-biased and cytotoxic T lymphocyte (CTL)-mediated cellular immune response. In addition, this formulation markedly elevated serum titers of HBsAg-specific IgG, IgG1, and IgG2a. **Conclusions**: These findings underscore the advantages of the LNP-PolyI:C adjuvant in enhancing both humoral and cellular immunity, demonstrating its considerable potential as a novel adjuvant.

## 1. Introduction

Vaccination is one of the most effective methods for controlling infectious diseases and a widely used intervention in public health practice [1]. Hepatitis B is an infectious disease caused by the hepatitis B virus (HBV). Long-term HBV infection can cause varying degrees of liver damage and may progressively develop into hepatitis, liver fibrosis, cirrhosis, and hepatocellular carcinoma [2]. Hepatitis B vaccination can effectively prevent hepatitis B, limit the progression of symptoms in carriers, and reduce the disease burden [3].

Recombinant protein and peptide subunit antigens typically exhibit weak immunogenicity and are often insufficient to induce robust protective humoral and cellular immune responses [4,5]. Consequently, most subunit antigens require co-formulation with adjuvants. Adjuvants are immunomodulatory substances added to vaccines that play a crucial role in regulating the host immune response, such as enhancing immunogenicity and reducing the required antigen dose [4,6]. Currently, the majority of licensed hepatitis B vaccines rely on aluminum-based adjuvants. Prominent examples include Recombivax HB (Merck & Co., USA), Engerix-B (GlaxoSmithKline Biologicals, USA), and several recombinant vaccines produced in China, such as those expressed in Saccharomyces cerevisiae (China National Biotec Group), *Hansenula polymorpha* (AIM Vaccine Co., Ltd.), and CHO cells (North China Pharmaceutical Company Ltd.) [6,7,8]. Aluminum adjuvants function through multiple mechanisms. They adsorb antigens via electrostatic interactions, creating a depot effect that slows antigen release and promotes the recruitment and accumulation of antigen-presenting cells (APCs) at the injection site. This process facilitates antigen uptake, processing, and presentation. In addition, aluminum adjuvant can activate dendritic cells. Following phagocytosis, it activates the NLRP3 inflammasome, leading to the release of active IL-1β and IL-18 [9]. Studies have shown that aluminum adjuvants can induce a Th2-biased immune response but are weak inducers of Th1 immune responses [9,10]. Therefore, aluminum adjuvants are suboptimal for viral diseases that rely on Th1-mediated immunity and the priming of cytotoxic CTLs. In addition, the recombinant hepatitis B vaccine Heplisav B, developed by Dynavax Technologies Corporation (USA), contains the CpG 1018 adjuvant. CpG 1018 is a negatively charged 22-base oligonucleotide that activates Toll-like receptor 9 (TLR9) and induces a Th1 immune response [4]. However, studies have reported that compared to adults receiving the Engerix-B, those receiving the Heplisav B exhibited higher reported rates of acute myocardial infarction, herpes zoster, and death [11]. These limitations underscore the need for novel adjuvant systems that can safely and effectively elicit both robust humoral and Th1-biased cellular immunity.

PolyI:C is a synthetic analog of double-stranded RNA (dsRNA) that functions as a pathogen-associated molecular pattern (PAMP) and activates multiple innate immune receptors located in endosomal and cytoplasmic compartments [12]. In antigen-presenting cells, endosomal PolyI:C is sensed by TLR3, whereas cytoplasmic PolyI:C engages RLRs, including RIG-I and melanoma differentiation–associated protein 5 (MDA-5) [13]. TLR3 signaling proceeds through the TIR-domain–containing adaptor-inducing interferon-β (TRIF), leading to activation of interferon regulatory factor 3 (IRF3), nuclear factor κB (NF-κB), and activator protein-1 (AP-1), which collectively drive the production of type I interferons and pro-inflammatory cytokines [14,15]. In contrast, RLR engagement triggers receptor oligomerization, exposure of the N-terminal caspase recruitment domain (CARD), and mitochondrial translocation. Subsequent signaling through the mitochondrial antiviral signaling (MAVS) protein robustly induces type I and III interferons, which promote type 1 cytokine responses [13]. The resulting type I interferon milieu enhances natural killer cell activity, supports antigen-specific CD8^+^ T cell responses, and promotes dendritic cell maturation, thereby linking innate immune sensing of PolyI:C to the development of effective antiviral and adaptive immune responses [16]. However, when administered alone as a vaccine adjuvant, PolyI:C is rapidly degraded by ubiquitous nucleases and reaches the cytosol inefficiently [17]. This short half-life and poor intracellular access blunt its immunopotentiating activity, while also requiring higher doses that can trigger systemic hyperinflammation. These higher doses increase the risk of excessive immune activation and autoimmunity [18,19]. Therefore, to address these issues and enhance the stability and safety of PolyI:C, researchers have developed various delivery platforms, including polymer films [20], hydrogels [21,22], and various micro and nanoparticle systems [23,24]. Among these, LNPs have emerged as a particularly promising candidate due to their efficient encapsulation and cytosolic delivery capabilities [25,26].

LNPs are nanoparticles composed of ionizable cationic lipids, other lipid types, and encapsulated cargo, and they are recognized as the most mature delivery platform. The success of LNP-containing mRNA vaccines, such as Comirnaty and Spikevax, during the COVID-19 pandemic has underscored the immense potential of LNP technology [27,28]. All currently FDA-approved LNP formulations employ a four-component lipid composition: an ionizable cationic lipid, a neutral helper lipid, cholesterol, and a PEG-lipid conjugate [29,30]. The intracellular delivery efficiency of LNPs relies on the protonation of the ionizable cationic lipid in the acidic endosomal environment; this process disrupts endosomal membrane integrity, facilitating the release of the encapsulated cargo. Helper phospholipids provide structural support during LNP formation and may participate in endosomal escape processes associated with membrane fusion. Cholesterol modulates membrane rigidity and integrity, contributing to particle structural stability while regulating in vivo delivery efficiency and organ distribution tendencies. Surface PEGylation inhibits protein adsorption and particle aggregation through steric hindrance, thereby prolonging circulation time and ensuring uniform particle size [30,31,32]. Based on the aforementioned structural and functional characteristics of LNPs, encapsulating PolyI:C within lipid nanoparticle systems can effectively protect it from nuclease degradation, thereby fully unleashing its potential as a vaccine adjuvant.

In this study, a detailed and systematic immunogenicity evaluation of the LNP-PolyI:C adjuvant was conducted in a mouse model, including the measurement of serum antibody levels and cellular immune cytokines. Our results provide a theoretical basis for the design of LNP adjuvants for specific vaccine applications. Herein, we demonstrate that LNP encapsulation significantly potentiates the innate immunostimulatory activity of PolyI:C compared to either soluble PolyI:C or empty LNPs alone. In vivo, intramuscular co-administration of recombinant HBsAg with LNP-encapsulated PolyI:C in mice elicited antigen-specific antibody titers surpassing those induced by soluble PolyI:C, while concurrently augmenting cellular immune responses.

## 2. Materials and Methods

### 2.1. LNP Encapsulating PolyI:C and Vaccine Preparation

The HBsAg and PolyI:C used in this study were both prepared in-house. HBsAg was expressed in *Pichia pastoris* and subsequently purified. PolyI:C was formed by annealing high-molecular-weight polyinosinic acid and polycytidylic acid under specific salt concentration conditions, followed by sterile filtration and aliquoting for storage. LNP-PolyI:C was synthesized by CATUG Biotechnology (Suzhou) Co., Ltd. (Suzhou, China) Briefly, PolyI:C was encapsulated into lipid nanoparticles using the hydrodynamic focusing (HDF) technique, as described by Singh et al. [33]. The lipid components comprising DOTAP:DSPC:Cholesterol:DSPE-PEG2000 were dissolved in ethanol at a molar ratio of 3:2:2:0.3. Subsequently, the lipid mixture was combined with a phosphate-buffered saline (PBS) solution containing PolyI:C at a defined volume ratio using a microfluidic mixer (INano L^+^, Micro & Nano, Shanghai, China). After ethanol removal and filtration, the encapsulation efficiency of PolyI:C by LNPs was determined using a fluorescence staining method. In parallel, an empty LNP without PolyI:C was prepared as a control. The LNP-PolyI:C mixture was characterized for its Z-average size and polydispersity index (PDI) using a Zetasizer Lab instrument (Malvern Panalytical, Malvern, UK). Upon arrival of the synthetic samples, animal experiments were carried out immediately. The remaining samples were stored at 2–8 °C for subsequent stability monitoring. The compositions of the designed vaccines are shown in Table 1.

### 2.2. Animal Experiments

Female Kunming mice (13–16 g), specific pathogen-free (SPF), were purchased from Liaoning Changsheng Biotechnology Co., Ltd. (Benxi, China). Upon arrival at the institutional animal facility, the mice were housed in a barrier environment (temperature: 20–25 °C; humidity: 40–70%; 12 h:12 h light-dark cycle) and fed a standard maintenance diet. After an acclimatization period of 3–5 days, the mice were randomly assigned to 5 groups (*n* = 8 per group) before immunization. According to the vaccine groups outlined in Table 1, each mouse was administered 100 µL of vaccine via intramuscular (i.m.) injection on days 0 and 14. Blood samples were collected from the retro-orbital venous plexus on days 14 and 28, and serum was separated for ELISA. On day 28, the mice were euthanized, and spleens were harvested for subsequent flow cytometry and ELISpot analysis.

### 2.3. Enzyme-Linked Immunosorbent Assay

ELISA was established to determine the levels of HBsAg-specific total IgG, IgG1, and IgG2a antibodies in the immune sera. The experimental procedure is briefly outlined as follows: Initially, 96-well ELISA plates were coated with HBsAg at a concentration of 2 μg/mL (100 μL per well) and left overnight at 4 °C. After coating, the plates were blocked with 3% bovine serum albumin in PBS (3% BSA-PBS) for 1 h at 37 °C. Next, serially diluted serum samples (prepared in 1.5% BSA-PBS) were added and incubated for 1 h at room temperature. Following that, HRP-conjugated secondary antibodies—including goat anti-mouse IgG (H + L) (Gene-Protein Link, Cat# P03S01, Beijing, China), IgG1 (SouthernBiotech, Cat# 1070-05, Birmingham, AL, USA), and IgG2a (SouthernBiotech, Cat# 1080-05, Birmingham, AL, USA)—all diluted in 1.5% BSA-PBS, were added and incubated at 37 °C for another hour. Subsequently, 100 μL of TMB substrate solution (Solarbio, Cat# PR1200, Beijing, China) was dispensed into each well, followed by a 10 min incubation at 37 °C. The enzymatic reaction was stopped by adding 50 μL of 2 M sulfuric acid. Between each step, the plates were washed three times with PBS containing 0.05% Tween-20. Absorbance readings were taken immediately at 450 nm and 630 nm using a Synergy H1 microplate reader (BioTek, Winooski, VT, USA), with blank wells serving as a zero reference. A sample was considered positive if its OD450 value exceeded that of the negative control by at least 2.1.

### 2.4. Isolation of Splenocytes

Splenocytes were prepared under sterile conditions using a laminar flow hood. Briefly, a 70 μm cell strainer (BD, Franklin Lakes, NJ, USA) was secured over a 50 mL centrifuge tube, and the spleen was placed inside the strainer. By gentle grinding and continuous addition of 10 mL PBS, cells were flushed into the tube. The suspension was then centrifuged; the supernatant was removed, and the pellet was treated with 5 mL of 1× RBC lysis buffer (BioLegend, Cat# 420301, San Diego, CA, USA) at 4 °C for 3 min. To stop lysis, 15 mL PBS was added. After a second centrifugation and removal of the supernatant, the remaining cells were washed once with 10 mL RPMI 1640 (Gibco, Cat# 11875-093, Waltham, MA, USA). The washed cell pellet was finally resuspended in RPMI 1640 supplemented with 10% fetal bovine serum (Gibco, Cat# 10099-141C, Waltham, MA, USA) and 1% penicillin-streptomycin (Solarbio, Cat# P7630, Beijing, China). Following counting, the concentration was adjusted to 5 × 10^6^ cells/mL.

### 2.5. Flow Cytometry Analysis

Multicolor flow cytometry was used to assess cytokine secretion by splenocytes. Unless otherwise specified, all incubations were carried out at 37 °C in 5% CO_2_; surface staining was performed at 4 °C in the dark, and intracellular staining at room temperature in the dark. Briefly, 800 µL of cell suspension was added to a 24-well plate and co-cultured overnight with the HBsAg peptide pool. Brefeldin A solution (Biolegend, Cat# San Diego, CA, 420601, USA) was then added for an additional 4 h to block cytokine release. After washing with PBS, cells were sequentially labeled with the Zombie NIR Fixable Viability Kit (Biolegend, Cat# 423105, San Diego, CA, USA) and blocked with TruStain FcX PLUS (Biolegend, Cat# 156603, San Diego, CA, USA). Surface markers were stained for 30 min with FITC anti-CD3, PerCP-Cy5.5 anti-CD4, and AF700 anti-CD8a. Following fixation and permeabilization, intracellular cytokines were detected by 30 min incubation with PE-Cy7 anti-TNF-α, APC anti-IFN-γ, PE anti-IL-6, BV605 anti-IL-4, and BV421 anti-IL-2. After washing with permeabilization buffer, cells were washed with PBS and acquired on a BD FACSCelesta flow cytometer (BD, Franklin Lakes, NJ, USA). Data were analyzed using FlowJo software v10.8.1 (BD, Franklin Lakes, NJ, USA).

### 2.6. Enzyme-Linked Immunospot Assay

A FluoroSpot assay was performed using the FluoroSpot Plus: Mouse IFN-γ/IL-4 kit (MabTech, Cat# FSP-3146-10, Nacka, Sweden) according to the manufacturer’s protocol. Briefly, 96-well plates were washed with sterile PBS and blocked with cell culture medium. Following this, 100 μL of cell suspension was added to each well. The wells were then stimulated with 100 μL of HBsAg peptide pool (test samples), phytohemagglutinin (DAKEWE, Cat# 2030411, Shenzhen, China) (positive control), or cell culture medium (negative control). All conditions were set up in triplicate. The plates were incubated in a cell culture incubator at 37 °C with 5% CO_2_ for 24 h. After incubation, the cell culture supernatant was discarded, and the plates were processed according to the kit protocol, which involved sequential incubation with detection monoclonal antibodies, fluorophore-conjugated secondary antibodies, and FluoroSpot enhancer solution. Finally, the plates were left to dry overnight at room temperature in the dark. The number of fluorescent spots in each well was detected using the Mabtech IRIS™ 2 instrument (MabTech, Nacka, Sweden), and the resulting data were exported for analysis. The mean number of spots ± SEM in triplicate wells was calculated and expressed as spots forming cells (SFCs) per 10^6^ splenocytes.

### 2.7. Statistical Analysis

Data are shown as mean ± SEM. Statistical evaluation was carried out with GraphPad Prism 9 (GraphPad Software, Boston, MA, USA). Group differences were assessed by one-way ANOVA followed by Tukey’s HSD test. Statistical significance was defined as *p* < 0.05. In one-way ANOVA, the R-squared value equals the effect size eta-squared (η^2^), which represents the proportion of total variation explained by between-group variation. 95% confidence intervals for mean differences are provided in the Supplementary File S1.

## 3. Results

### 3.1. Stability Results of LNP-PolyI:C

**Figure 1:**
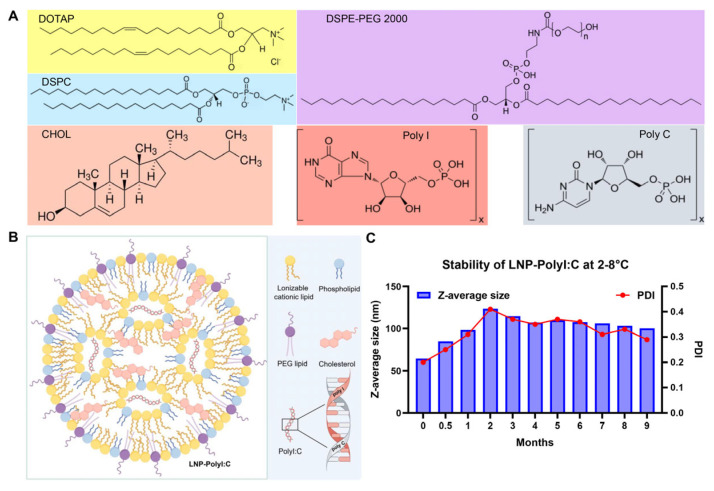
Composition and stability results of LNP-PolyI:C. (**A**) Chemical structures of the components constituting LNP-PolyI:C. (**B**) Schematic diagram of the LNP-PolyI:C structure. The figure was created with Figdraw (www.figdraw.com). (**C**) The colloidal stability of LNP-PolyI:C stored at 2–8 °C for 9 months. DOTAP: 1,2-stearoyl-3-trimethylammonium-propane, DSPC: 1,2-distearoyl-sn-glycero-3-phosphocholine, CHOL: cholesterol, DSPE-PEG 2000: 1,2-distearoyl-sn-glycero-3-phosphoethanolamine-N-[methoxy(polyethylene glycol)-2000].

Following the method reported by Singh et al. [33], PolyI:C was encapsulated into LNPs using the HDF technique. The lipid components (containing DOTAP, DSPC, CHOL, and DSPE-PEG 2000) dissolved in ethanol, and a PolyI:C solution dissolved in PBS were mixed (Figure 1A,B). The colloidal stability of LNP-PolyI:C stored at 2–8 °C for 9 months was monitored using DLS on a Zetasizer Lab instrument. As shown in Figure 1C, the Z-average size and PDI of LNP-PolyI:C exhibited dynamic changes characterized by an initial increase followed by stabilization. Both parameters peaked at the 2-month time point (123 nm and 0.41, respectively) and then plateaued. In addition, the encapsulation efficiency of PolyI:C by LNP exceeded 90%, indicating that this encapsulation process possesses excellent drug-loading efficiency.

### 3.2. The LNP-PolyI:C Adjuvant Significantly Enhances Serum Antibody Response Levels

**Figure 2:**
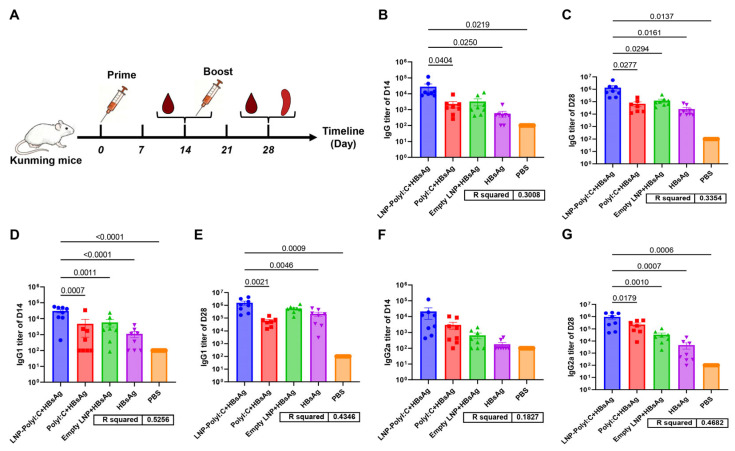
Detection of HBsAg-specific antibody expression in mouse serum by ELISA. (**A**) Schematic diagram of the mouse immunization protocol. (**B**) Levels of HBsAg-specific total IgG, (**D**) IgG1, and (**F**) IgG2a antibodies induced in serum from each treatment group on day 14 (D14) after the primary immunization. (**C**) Levels of HBsAg-specific total IgG, (**E**) IgG1, and (**G**) IgG2a antibodies induced in serum from each treatment group on day 14 (D28) after the secondary immunization. Antibody titers in all negative control groups (PBS group) were set to 100. Data represent mean ± SEM. Multiple comparisons were performed using one-way analysis of variance (ANOVA) followed by Tukey’s honestly significant difference (HSD) test. *p* < 0.05 was considered statistically significant. The experiment was conducted with biological replicates. The sample size (*n*) for all groups was 8 on day 14. On day 28, *n* = 7 for the PolyI:C + HBsAg group and *n* = 8 for all other groups.

To evaluate the ability of the LNP-PolyI:C adjuvant to induce neutralizing antibodies, an immunogenicity study of the LNP-PolyI:C/HBsAg vaccine was conducted in mice. The experimental procedure is shown in Figure 2A. Animals were immunized according to a 0- and 14-day schedule, with each mouse receiving 4 μg of HBsAg by intramuscular injection at each dose. PBS injection served as the negative control. The study consisted of five groups: LNP-PolyI:C + HBsAg, PolyI:C + HBsAg, empty LNP + HBsAg, HBsAg alone, and PBS. Blood samples were collected from the retro-orbital venous plexus on day 14 (D14) and day 28 (D28) after the primary immunization. ELISA measured serum levels of HBsAg-specific total IgG, IgG1, and IgG2a antibodies. The results demonstrated that the LNP-PolyI:C adjuvant group induced a robust early humoral immune response as early as day 14 post-primary immunization. The IgG1 titer in this group was significantly higher than that in the other four groups. The total IgG titer was significantly higher than that in the PolyI:C + HBsAg group, the HBsAg-alone group, and the PBS group, but no statistically significant difference was observed compared to the empty LNP + HBsAg group (Figure 2B). Although the IgG2a titer did not differ significantly from that in the other four groups, it was the highest among all groups (Figure 2F). On day 14 post-booster immunization (D28), all vaccinated groups, except the negative control, mounted a robust anamnestic response, with total IgG, IgG1, and IgG2a titers exceeding those observed after primary immunization. The levels of all three HBsAg-specific antibodies in the LNP-PolyI:C adjuvant group were significantly higher than those in the other four groups, with only IgG1 showing no statistically significant difference compared to the Empty LNP + HBsAg group (Figure 2C,E,G).

### 3.3. The LNP-PolyI:C Adjuvant Significantly Enhances Cytokine Expression In Vivo

**Figure 3:**
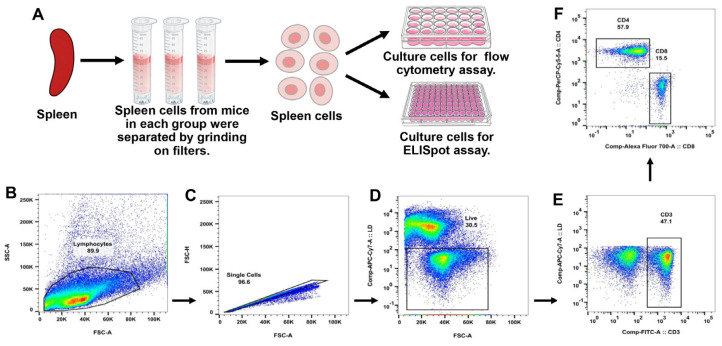
Expression profiles of various immune cells in mouse splenocytes stimulated with an HBsAg peptide library, as detected by flow cytometry. (**A**) Schematic diagram of mouse splenocyte isolation and immune stimulation. Pseudocolor plots show representative gating results close to the mean for (**B**) lymphocytes, (**C**) single cells, (**D**) live cells, (**E**) CD3^+^ T cells, and (**F**) CD4^+^/CD8^+^ T cells. The pseudocolor indicates the relative density of cell events, with blue representing low density and red representing high density.

**Figure 4:**
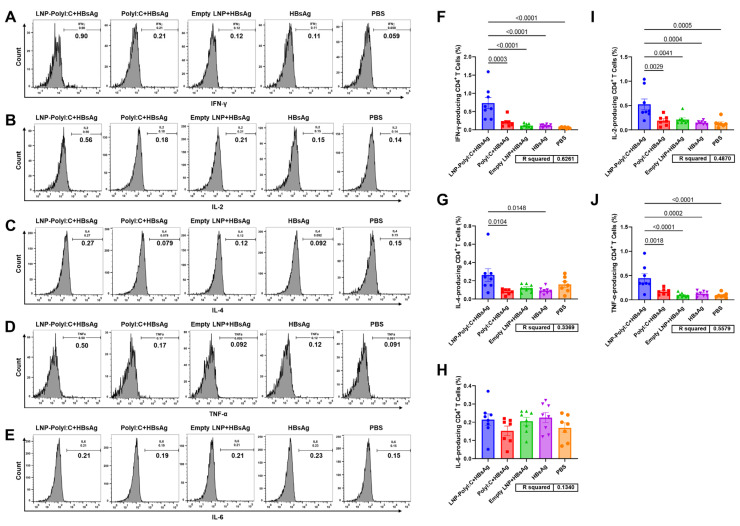
Detection of cytokine expression in CD4^+^ T cells from mouse splenocytes stimulated with an HBsAg peptide pool by flow cytometry. Histograms show gated representative results near the mean for the expression of (**A**) IFN-γ, (**B**) IL-2, (**C**) IL-4, (**D**) TNF-α, and (**E**) IL-6. (**F**–**J**) Quantitative analysis of each cytokine across groups. Data represent mean ± SEM. Multiple comparisons were performed using one-way analysis of variance (ANOVA) followed by Tukey’s honestly significant difference (HSD) test. *p* < 0.05 was considered statistically significant. The experiment was conducted with biological replicates. The sample size (*n*) for both the PolyI:C + HBsAg group and the PBS group was 7, and *n* = 8 for all other groups.

**Figure 5:**
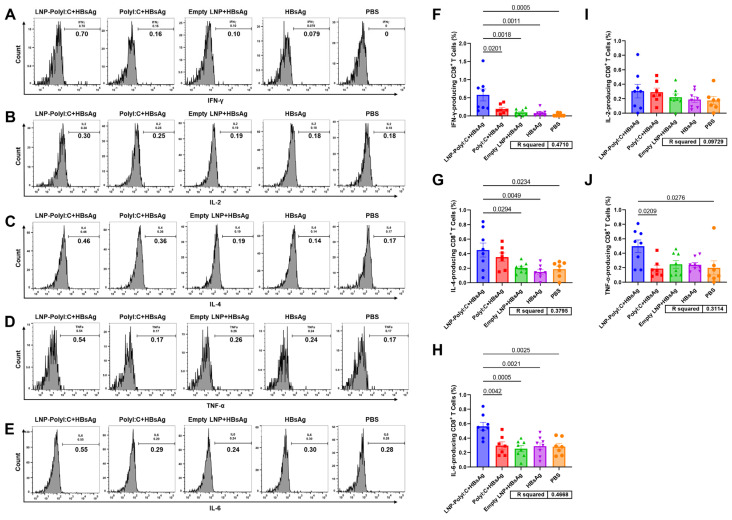
Detection of cytokine expression in CD8^+^ T cells from mouse splenocytes stimulated with an HBsAg peptide pool by flow cytometry. Histograms show gated representative results near the mean for the expression of (**A**) IFN-γ, (**B**) IL-2, (**C**) IL-4, (**D**) TNF-α, and (**E**) IL-6. (**F**–**J**) Quantitative analysis of each cytokine across groups. Data represent mean ± SEM. Multiple comparisons were performed using one-way analysis of variance (ANOVA) followed by Tukey’s honestly significant difference (HSD) test. *p* < 0.05 was considered statistically significant. The experiment was conducted with biological replicates. The sample size (*n*) for both the PolyI:C +HBsAg group and the PBS group was 7, and *n* = 8 for all other groups.

Given the critical role of Th1-type and CTL-type cellular immunity in HBV prevention, the ability of the LNP-PolyI:C adjuvant to induce T-cell responses was further evaluated. The experimental procedure is shown in Figure 3A. On day 14 post-boost immunization (D28), splenocytes were isolated and stimulated with a peptide pool covering the full length of HBsAg. In flow cytometry analysis, splenocytes were stained with multiple fluorescent antibodies and analyzed using a flow cytometer. A sequential gating strategy was employed to identify the target immune cell populations (Figure 3B–F). As shown in Figure 4 and Figure 5, the LNP-PolyI:C adjuvant group induced a robust HBsAg-specific T-cell response. The numbers of both IFN-γ^+^CD4^+^ and IFN-γ^+^CD8^+^ T cells produced in this group were significantly increased and markedly higher than those in the other four groups. In addition to IFN-γ, CD4^+^ T cells in the LNP-PolyI:C adjuvant group secreted a higher proportion of IL-2 and TNF-α, while CD8^+^ T cells secreted a higher proportion of IL-6. However, no significant differences in the numbers of IL-6^+^CD4^+^ and IL-2^+^CD8^+^ T cells were observed among the treatment groups. Furthermore, the numbers of IL-4^+^CD4^+^, IL-4^+^CD8^+^, and TNF-α^+^CD8^+^ T cells were also assessed. The results indicated that the number of IL-4^+^CD4^+^ T cells in the LNP-PolyI:C + HBsAg group was significantly higher than that in the PolyI:C + HBsAg and HBsAg alone groups, but no significant difference was observed compared to the empty LNP + HBsAg and PBS groups. The proportion of IL-4^+^CD8^+^ T cells in the LNP-PolyI:C + HBsAg group was comparable to that in the PolyI:C + HBsAg group and was significantly higher than that in the other three groups. The number of TNF-α^+^CD8^+^ T cells in the LNP-PolyI:C + HBsAg group was significantly higher than that in the PolyI:C + HBsAg and PBS groups, with no significant difference compared to the empty LNP + HBsAg and HBsAg alone groups.

**Figure 6:**
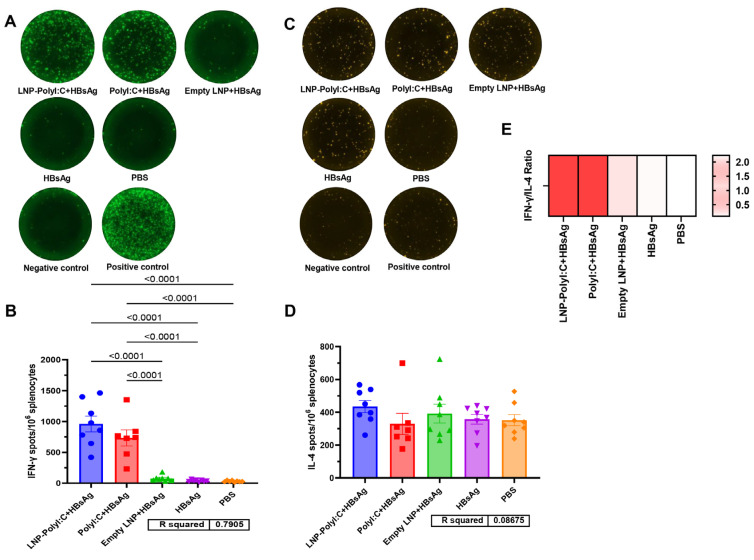
Detection of cytokine expression in mouse splenocytes stimulated with an HBsAg peptide library by Enzyme-Linked Immunospot (ELISpot). (**A**) Representative spot images of IFN-γ-secreting splenocytes and (**B**) quantitative analysis results for each group. (**C**) Representative spot images of IL-4-secreting splenocytes and (**D**) quantitative analysis results for each group. (**E**) The ratio of the average spot counts for IFN-γ to IL-4 in each group. Data represent mean ± SEM. Multiple comparisons were performed using one-way analysis of variance (ANOVA) followed by Tukey’s honestly significant difference (HSD) test. *p* < 0.05 was considered statistically significant. The experiment was conducted with biological replicates. The sample size (*n*) for the PolyI:C + HBsAg group was 7, and *n* = 8 for all other groups.

In addition, HBsAg-specific IFN-γ/IL-4 secreting T cells were measured by ELISpot assay in this study. As shown in Figure 6, significant differences in the number of HBsAg-specific IFN-γ spots were observed among the groups. The LNP-PolyI:C + HBsAg group exhibited the highest number of SFCs per 10^6^ splenocytes (959.5 ± 127.3), followed by the PolyI:C + HBsAg group (733.3 ± 130.3). Although no statistically significant difference was observed between these two PolyI:C-containing groups, both were significantly higher than the empty LNP + HBsAg group (76.4 ± 16.6), the HBsAg-alone group (38.4 ± 5.8), and the PBS group (30.9 ± 4.4). However, no significant differences in the number of IL-4 spots were observed among the groups. The number of SFCs per 10^6^ splenocytes for each group was as follows: LNP-PolyI:C + HBsAg group (434.8 ± 36.7), PolyI:C + HBsAg group (329.4 ± 64.6), empty LNP + HBsAg alone group (391.9 ± 57.3), HBsAg group (357.4 ± 29.8), and PBS group (352.1 ± 33.7).

## 4. Discussion

Recombinant subunit antigens often require adjuvants to elicit protective immunity, yet conventional options such as alum primarily induce Th2 responses. PolyI:C, a dsRNA analog, activates both endosomal TLR3 and RLRs, thereby promoting robust Th1 and cytotoxic T lymphocyte responses, which are crucial for antiviral and anticancer vaccines. However, soluble PolyI:C suffers from nuclease degradation, poor cellular uptake, and inability to access cytoplasmic RLRs. To overcome these limitations, we propose encapsulating PolyI:C within LNPs. Drawing on the established delivery mechanisms of mRNA vaccines (protecting nucleic acids, enhancing uptake by antigen-presenting cells, and promoting endosomal escape), LNPs can achieve efficient delivery of PolyI:C into the cytoplasm. Against this background, this study constructed an LNP-PolyI:C adjuvant system and evaluated its potential as an adjuvant for hepatitis B vaccines.

Humoral immunity, particularly the induction of high-titer, high-affinity neutralizing antibodies, constitutes the core mechanism by which hepatitis B vaccines prevent infection. Following the entry of vaccine antigens into the body, antigen-presenting cells secrete distinct cytokines that guide the polarization of naïve CD4^+^ T cells toward either a Th1 or Th2 phenotype [34]. Th1 cells secrete IFN-γ, which drives B cells to produce IgG2a subtype antibodies. This subtype exhibits strong complement-activating capacity and high affinity for Fc receptors, thereby effectively mediating opsonophagocytosis and the clearance of intracellular pathogens. In contrast, Th2 cells secrete IL-4, promoting B-cell class-switching toward the IgG1 subtype, which primarily exerts neutralizing activity but possesses relatively weak effector functions [35,36]. In this study, the LNP-PolyI:C adjuvant elicited high-titer, high-affinity IgG and IgG1 antibodies as early as D14. By D28, antibody titers had further increased, accompanied by a significant rise in IgG2a levels. These results demonstrate the advantage of the LNP-PolyI:C adjuvant in optimizing the humoral immune response of the hepatitis B vaccine.

Th1 and Th2 cells form a regulatory network through the mutual antagonism of IFN-γ and IL-4, which directly determines the intensity of vaccine-induced IgG2a and IgG1 responses [37]. Therefore, the precise modulation of Th1/Th2 polarization via adjuvant selection represents a rational strategy for tailoring vaccine-induced immunity to achieve optimal protective efficacy. The primary contribution of PolyI:C was to enhance the Th1-type IgG2a response and total antibody titers, thereby promoting T cell-dependent antibody affinity maturation. The data from this study support this mechanism: following the addition of PolyI:C, IgG2a levels on both D14 and D28 showed an increasing trend, confirming the directional driving effect of PolyI:C on Th1 polarization.

T lymphocytes are the key effector cells of cellular immunity, comprising primarily CD4^+^ and CD8^+^ T cell subsets. Th1 cells produce cytokines such as IFN-γ, IL-2, and TNF-α, which promote macrophage activation and recruitment, facilitate CTL differentiation, and induce cellular immune responses. In contrast, Th2 cells produce cytokines, including IL-4 and IL-6, which promote B cell proliferation and differentiation and elicit humoral immune responses. CD8^+^ T cells, also known as CTLs, serve as the terminal effectors of cellular immunity. They similarly secrete IFN-γ and TNF-α and directly eliminate infected target cells via the perforin/granzyme pathway or death receptor-mediated pathways [36,38]. In this study, the LNP-PolyI:C/HBsAg vaccine significantly induced HBsAg-specific Th1-type CD4^+^ T-cell responses (IFN-γ^+^, TNF-α^+^, and IL-2^+^) and CTL-type CD8^+^ T-cell responses (IFN-γ^+^, IL-6^+^). This indicates that the LNP-PolyI:C adjuvant system synergistically activates cellular immunity through the dual mechanisms of Th1 and CTL. This finding is consistent with the study by Liu et al. [39] on an HBV vaccine containing LNP-PolyI:C, which also reported significant induction of IFN-γ^+^CD8^+^ T cells.

Notably, in the ELISpot results, the number of IFN-γ SFCs in the LNP-PolyI:C group was approximately 31% higher than that in the PolyI:C group. This suggests that LNP encapsulation facilitates intracellular delivery and endosomal escape of PolyI:C, thereby further amplifying T-cell activation via the relevant signaling pathway. Although the number of IFN-γ SFCs in the empty LNP adjuvant group was slightly higher than that in the antigen-alone group, it remained substantially lower than that in the PolyI:C-containing groups. This indicates that the LNP carrier itself has a limited enhancing effect on cellular immunity, whereas PolyI:C is the key determinant for inducing potent Th1/CTL responses. In addition, there was no significant difference in the secretion level of IL-4 among the groups. Nevertheless, the IFN-γ/IL-4 ratio remained significantly higher in all PolyI:C-containing groups compared to other groups, confirming that PolyI:C induces an immune response characterized by a Th1 bias.

As a key component in enhancing the immune response, the stability of an adjuvant’s physicochemical properties directly determines its immunopotentiating effect in vaccines. In this study, DLS analysis revealed that the Z-average particle size and PDI of LNP-PolyI:C increased initially before plateauing, suggesting that the particles may have undergone significant aggregation/fusion processes in the early stage, after which the system gradually reached a kinetically stable state. It has been reported that when lipid carriers are used for drug delivery, the PDI is typically kept below 0.3 to maintain a homogeneous population of phospholipid vesicles [40]. However, in this study, the PDI of LNP-PolyI:C exceeded 0.3 after one month of storage at 2–8 °C, indicating that further optimization of its formulation is necessary to control the increase in PDI during long-term storage. It should be noted that the animal experiments in this study were initiated immediately upon receipt of the LNP-PolyI:C samples, at which time the PDI was approximately 0.2. Given that this study did not investigate the differences in the immunogenicity of LNP-PolyI:C in animals after two months of storage, its adjuvant effect may change with prolonged storage, and the potential implications for vaccine development warrant further exploration. In subsequent development plans, in addition to optimizing the LNP-PolyI:C formulation and evaluating its performance changes after long-term storage, we will also investigate whether the increase in PDI originates from the LNP carrier itself. Furthermore, multiple studies have demonstrated that lyophilization can enhance the stability of mRNA-LNP vaccines [41,42,43]. Therefore, subsequent research will also explore the application of lyophilization technology to LNP-PolyI:C/HBsAg vaccines to improve storage stability and facilitate their clinical translation.

## 5. Conclusions

In this study, a polyinosinic-polycytidylic acid adjuvant system based on lipid nanoparticles was successfully developed, and its immunogenicity and stability as a hepatitis B vaccine adjuvant were systematically evaluated. This adjuvant system significantly enhanced the immunogenicity of the hepatitis B vaccine, and the delivery efficiency of PolyI:C was significantly enhanced following LNP encapsulation. Mouse immunization experiments demonstrated that the LNP-PolyI:C adjuvant induced a strong humoral and cellular immune response: serum levels of total IgG, IgG1, and IgG2a were significantly elevated; meanwhile, the Th1 and CTL responses in splenocytes were significantly enhanced. This immune profile contrasted sharply with the significant Th2 bias observed with aluminum adjuvants. In summary, the LNP-PolyI:C adjuvant holds considerable application potential for the prevention or control of viral infections, such as HBV, where synergistic cellular immunity is required for viral clearance.

## Figures and Tables

**Table 1:** The compositions of the vaccines.

Vaccine Group	Vaccine Ingredients (per Milliliter)			
	HBsAg (μg)	LNP-PolyI:C (μg)	PolyI:C (μg)	Empty LNP
LNP-PolyI:C + HBsAg	40	1000		
PolyI:C + HBsAg	40		1000	
Empty LNP + HBsAg	40			The volume of the empty LNP is the same as that of LNP-PolyI:C.
HBsAg	40			
PBS				

## Data Availability

The dataset is available on request from the authors.

## Supplementary Materials

The following supporting information can be downloaded at: https://www.mdpi.com/article/10.3390/vaccines14050397/s1, File S1: 95% confidence intervals for mean differences for Figure 2, Figure 4, Figure 5 and Figure 6.

## Abbreviations

The following abbreviations are used in this manuscript:

PolyI:C	Polyinosinic-polycytidylic acid
LNP	Lipid nanoparticle
HBsAg	Hepatitis B surface antigen
ELISA	Enzyme-linked immunosorbent assay
ELISpot	Enzyme-linked immunospot
CTL	Cytotoxic T lymphocyte
HBV	Hepatitis B virus
APC	Antigen-presenting cell
TLR9	Toll-like receptor 9
dsRNA	Double-stranded RNA
PAMP	Pathogen-associated molecular pattern
MDA-5	Melanoma differentiation–associated protein 5
IRF3	Interferon regulatory factor 3
NF-κB	Nuclear factor κB
AP-1	Activator protein-1
CARD	Caspase recruitment domain
MAVS	Mitochondrial antiviral signaling
PBS	Phosphate-buffered saline
PDI	Polydispersity index
SPF	Specific pathogen-free
BSA	Bovine serum albumin
SFC	Spots forming cell
ANOVA	One-way analysis of variance
HSD	Honestly significant difference
HDF	Hydrodynamic focusing
DLS	Dynamic light scattering
DOTAP	1,2-stearoyl-3-trimethylammonium-propane
DSPC	1,2-distearoyl-sn-glycero-3-phosphocholine
DSPE-PEG 2000	1,2-distearoyl-sn-glycero-3-phosphoethanolamine-N-[methoxy(polyethylene glycol)-2000]
