# Supplementary material for: Lipid Nanoparticle-Encapsulated PolyI:C as an Adjuvant Enhances Both Humoral and Cellular Immune Responses to the Hepatitis B Vaccine
Source: Vaccines (Basel). 2026 Apr 29;14(5):397. doi: 10.3390/vaccines14050397 (PMC13211435; doi:10.3390/vaccines14050397)
Supplement: Supplementary file 1 [file vaccines-14-00397-s001.zip › vaccines-4253479-supplementary.pdf]

File S1. 95% confidence intervals for mean differences for Figures 2 and 4–6.

Figure 2: 95.00% CI of diff.

**Tukey's multiple comparisons test**	**IgG titer of D14**	**IgG titer of D28**	**IgG1 titer of D14**	**IgG1 titer of D28**	**IgG2a titer of D14**	**IgG2a titer of D28**
LNP-PolyI:C+HBsAg vs. PolyI:C+HBsAg	790.1 to 50795	102434 to 2471708	9182 to 41682	451591 to 2574360	-8277 to 44329	90560 to 1316452
LNP-PolyI:C+HBsAg vs. Empty LNP+HBsAg	-195.8 to 49809	89041 to 2377978	8139 to 40639	-9965 to 2040824	-5919 to 46686	305222 to 1489546
LNP-PolyI:C+HBsAg vs. HBsAg	2513 to 52517	184929 to 2473866	12902 to 45402	335619 to 2386409	-5440 to 47165	333900 to 1518224
LNP-PolyI:C+HBsAg vs. PBS	2970 to 52975	209989 to 2498926	13931 to 46431	548010 to 2598800	-5362 to 47244	338402 to 1522726
PolyI:C+HBsAg vs. Empty LNP+HBsAg	-25988 to 24017	-1238199 to 1131076	-17292 to 15208	-1558931 to 563838	-23945 to 28660	-419069 to 806823
PolyI:C+HBsAg vs. HBsAg	-23280 to 26725	-1142311 to 1226964	-12530 to 19970	-1213346 to 909423	-23466 to 29139	-390390 to 835502
PolyI:C+HBsAg vs. PBS	-22822 to 27182	-1117251 to 1252024	-11500 to 20999	-1000955 to 1121814	-23388 to 29218	-385888 to 840004
Empty LNP+HBsAg vs. HBsAg	-22294 to 27711	-1048581 to 1240356	-11487 to 21013	-679810 to 1370980	-25824 to 26782	-563484 to 620841
Empty LNP+HBsAg vs. PBS	-21837 to 28168	-1023521 to 1265416	-10458 to 22042	-467419 to 1583371	-25745 to 26860	-558982 to 625343
HBsAg vs. PBS	-24545 to 25460	-1119409 to 1169528	-15221 to 17279	-813004 to 1237786	-26224 to 26381	-587660 to 596664

Figure 4: 95.00% CI of diff.

Tukey's multiple comparisons test	IFN-γ-producing CD4+ T Cells (%)	IL-4-producing CD4+ T Cells (%)	IL-6-producing CD4+ T Cells (%)	IL-2-producing CD4+ T Cells (%)	TNF-α-producing CD4+ T Cells (%)
LNP-PolyI:C+HBsAg vs. PolyI:C+HBsAg	0.2099 to 0.8376	0.03345 to 0.3344	-0.04981 to 0.1730	0.09286 to 0.5743	0.08577 to 0.4696
LNP-PolyI:C+HBsAg vs. Empty LNP+HBsAg	0.3157 to 0.9220	-0.002145 to 0.2886	-0.09900 to 0.1162	0.07995 to 0.5450	0.1620 to 0.5328
LNP-PolyI:C+HBsAg vs. HBsAg	0.3187 to 0.9250	0.02536 to 0.3161	-0.1187 to 0.09650	0.1450 to 0.6100	0.1327 to 0.5035
LNP-PolyI:C+HBsAg vs. PBS	0.3625 to 0.9901	-0.04598 to 0.2550	-0.06638 to 0.1564	0.1481 to 0.6296	0.1591 to 0.5429
PolyI:C+HBsAg vs. Empty LNP+HBsAg	-0.2187 to 0.4089	-0.1912 to 0.1098	-0.1644 to 0.05844	-0.2618 to 0.2196	-0.1222 to 0.2616
PolyI:C+HBsAg vs. HBsAg	-0.2157 to 0.4119	-0.1637 to 0.1373	-0.1841 to 0.03869	-0.1968 to 0.2846	-0.1515 to 0.2324
PolyI:C+HBsAg vs. PBS	-0.1715 to 0.4767	-0.2349 to 0.07600	-0.1316 to 0.09848	-0.1933 to 0.3039	-0.1249 to 0.2715
Empty LNP+HBsAg vs. HBsAg	-0.3002 to 0.3062	-0.1179 to 0.1729	-0.1274 to 0.08787	-0.1675 to 0.2975	-0.2147 to 0.1562
Empty LNP+HBsAg vs. PBS	-0.2564 to 0.3712	-0.1892 to 0.1118	-0.07501 to 0.1478	-0.1644 to 0.3171	-0.1883 to 0.1955
HBsAg vs. PBS	-0.2594 to 0.3682	-0.2167 to 0.08427	-0.05526 to 0.1675	-0.2294 to 0.2521	-0.1591 to 0.2247

Figure 5: 95.00% CI of diff.

Tukey's multiple comparisons test	IFN-γ-producing CD8+ T Cells (%)	IL-4-producing CD8+ T Cells (%)	IL-6-producing CD8+ T Cells (%)	IL-2-producing CD8+ T Cells (%)	TNF-α-producing CD8+ T Cells (%)
LNP-PolyI:C+HBsAg vs. PolyI:C+HBsAg	0.04559 to 0.7362	-0.1378 to 0.3350	0.06784 to 0.4657	-0.2388 to 0.2702	0.03478 to 0.5840
LNP-PolyI:C+HBsAg vs. Empty LNP+HBsAg	0.1500 to 0.8172	0.01786 to 0.4746	0.1164 to 0.5008	-0.1609 to 0.3309	-0.01468 to 0.5159
LNP-PolyI:C+HBsAg vs. HBsAg	0.1692 to 0.8363	0.07298 to 0.5298	0.08167 to 0.4661	-0.1300 to 0.3618	-0.007803 to 0.5228
LNP-PolyI:C+HBsAg vs. PBS	0.2079 to 0.8985	0.02616 to 0.4990	0.08069 to 0.4786	-0.1248 to 0.3842	0.02392 to 0.5732
PolyI:C+HBsAg vs. Empty LNP+HBsAg	-0.2526 to 0.4380	-0.08873 to 0.3841	-0.1571 to 0.2408	-0.1852 to 0.3238	-0.3334 to 0.2158
PolyI:C+HBsAg vs. HBsAg	-0.2334 to 0.4572	-0.03360 to 0.4392	-0.1919 to 0.2060	-0.1544 to 0.3547	-0.3265 to 0.2227
PolyI:C+HBsAg vs. PBS	-0.1943 to 0.5189	-0.08016 to 0.4082	-0.1926 to 0.2183	-0.1489 to 0.3769	-0.2945 to 0.2728
Empty LNP+HBsAg vs. HBsAg	-0.3145 to 0.3527	-0.1733 to 0.2835	-0.2270 to 0.1575	-0.2150 to 0.2768	-0.2584 to 0.2722
Empty LNP+HBsAg vs. PBS	-0.2758 to 0.4149	-0.2201 to 0.2527	-0.2279 to 0.1700	-0.2098 to 0.2992	-0.2267 to 0.3225
HBsAg vs. PBS	-0.2949 to 0.3957	-0.2752 to 0.1976	-0.1932 to 0.2047	-0.2407 to 0.2684	-0.2336 to 0.3157

Figure 6: 95.00% CI of diff.

Tukey's multiple comparisons test	IFN-γ spots/10^6 splenocytes	IL-4 spots/10^6 splenocytes
LNP-PolyI:C+HBsAg vs. PolyI:C+HBsAg	-101.0 to 553.5	-83.83 to 294.5
LNP-PolyI:C+HBsAg vs. Empty LNP+HBsAg	567.0 to 1199	-139.9 to 225.6
LNP-PolyI:C+HBsAg vs. HBsAg	605.0 to 1237	-105.4 to 260.1
LNP-PolyI:C+HBsAg vs. PBS	612.5 to 1245	-100.1 to 265.4
PolyI:C+HBsAg vs. Empty LNP+HBsAg	329.6 to 984.2	-251.6 to 126.7
PolyI:C+HBsAg vs. HBsAg	367.6 to 1022	-217.1 to 161.2
PolyI:C+HBsAg vs. PBS	375.1 to 1030	-211.8 to 166.5
Empty LNP+HBsAg vs. HBsAg	-278.2 to 354.2	-148.2 to 217.2
Empty LNP+HBsAg vs. PBS	-270.7 to 361.7	-143.0 to 222.5
HBsAg vs. PBS	-308.7 to 323.7	-177.5 to 188.0
